# How Sharp Objects Injuries Impact our Healthcare Workers: Unveiling Perspective and Preventive Imperatives

**DOI:** 10.7759/cureus.56524

**Published:** 2024-03-20

**Authors:** Ethar N Ibrahim, Soha Kannan, Laith Al Habahbeh, Omar H Makhamreh, Eman Khreisat, Martin KAKICH, Issa Khoury, Mohammad Abu Kaff, Amro Odah, Anees Hjazeen, Saif A Jabali, Rami Alqroom

**Affiliations:** 1 Anaesthesia, Jordanian Royal Medical Services, Irbid, JOR; 2 Health Policy and Nursing, Directorate of Royal Medical Services Journal, Amman, JOR; 3 Radiation Oncology, King Hussein Medical Center, Amman, JOR; 4 General Surgery, Jordanian Royal Medical Services, Amman, JOR; 5 Family Medicine, King Hussein Medical Center, Amman, JOR; 6 Neurosurgery, King Hussein Medical Center, Amman, JOR; 7 Neurological Surgery, King Hussein Medical Center, Amman, JOR; 8 Pediatric Neurology, Queen Alia Hospital, Amman, JOR; 9 Community Health, Nursing, and Biostatistics, Jordanian Royal Medical Services, Amman, JOR; 10 Community Medicine, Jordanian Royal Medical Services, Amman, JOR

**Keywords:** hepatitis b, hiv prophylaxis, healthcare workers, occupational hazard, bloodborne pathogens, sharp object injuries

## Abstract

Introduction

Sharp object injuries in the medical field present a considerable occupational hazard for healthcare workers (HCWs), encompassing a spectrum of consequences from immediate discomfort to enduring health consequences. These injuries may expose HCWs to potential infections. Despite efforts to control sharp object injuries in healthcare environments, they are present at every stage involving using or disposing of medical sharp instruments.

In Jordan, limited research has focused on sharp object injuries, with most data included from studies concentrating on practicing nurses or nursing students. Consequently, further research is necessary to comprehend the causes behind the high sharp object injury rate and the insufficient knowledge of safety practices and preventive guidelines.

Objectives

This study was conducted to investigate the impact of sharp object injuries on HCWs, underlying causes, and potential consequences causes of needlestick injuries. To highlight perspective and preventive imperatives.

Methods and patients

This retrospective institutional-based cross-sectional chart analysis was conducted by reviewing all sharp object injuries report sheets and extracting data directly from these reports for analysis. The study encompassed all reported cases occurring between 2018 and 2023. All the participants’ data handling was accomplished according to the Declaration of Helsinki (2013) and the Health Insurance Portability and Accountability (HIPAA) Acts.

Results

A total of 146 self-reported hospital workers were included in the study. Within the final cohort, 52.73% of the participants were male (77/146), with an average age at diagnosis of 38.6±7.87 years (ranging from 20 to 52 years). Conversely, females comprised 47.27% of the cohort population (69/146) and had an average age at diagnosis of 34.73±6.73 years (ranging from 19 to 47 years). The age group 20-29 years was the most prominent age group, statistical analysis of age and gender data revealed significant differences.

The overall prevalence of sharp object injuries was 11.83%, indicating that a sizable portion of HCWs is at risk of exposure to bloodborne pathogens. Among the different professional categories, Physicians constituted the majority of sharp object injuries reported victims in 41 cases (28.08%), followed by nurses in 38 cases (26.02%). Statistical analysis of the profession’s data revealed significant differences (*P*<0.001). Notably, sharp object injuries were most reported in wards.

The leading procedures that caused sharp object injuries were identified as during needle recapping in 53 instances (36.30%), then followed by medical waste treatment in 32 cases (21.92%). The left hand was the most affected body part, reported in 83 cases (56.84%). All injured individuals reported the incident promptly. No seroconversions were documented within the reviewed cases during the study period.

Conclusion

Injuries caused by sharp objects persist as a significant danger for hospital employees, posing immediate harm and long-term health risks linked to bloodborne pathogens.

The findings stress the continuous responsibility of healthcare institutions to prioritize staff safety by addressing the root causes of sharp object injuries and fostering reporting and prevention cultures. Underreporting reasons are diverse, encompassing factors like time constraints, fear of consequences, and the misconception of injury insignificance.

## Introduction

Sharp object injuries in the medical field present a considerable occupational hazard for healthcare workers (HCWs), encompassing a spectrum of consequences from immediate discomfort to enduring health consequences. These injuries may expose HCWs to the potential of over twenty various infections, including conditions like syphilis, malaria, herpes, hepatitis B, hepatitis C, and human immunodeficiency virus (HIV) [1-4]. 

Despite the World Health Organization’s (WHO) efforts to control sharp object injuries in healthcare environments, they are present at every stage involving using or disposing of medical sharp instruments [5-7]. Although the majority of sharp object injuries are reported in developing countries, in developed countries, where advanced prevention measures such as real-time injury monitoring systems and standardized operating protocols are in place, sharp object injuries continue to occur, and many reports coming from developed countries, which signifying that sharp objects injuries might be a global concern [8,9]. Globally, 32.4-44.5% of healthcare professionals report experiencing at least one accidental sharp injury or needle stick annually [1,2]. In the United States, an estimated 385,000 sharp object injuries occur among hospital healthcare workers annually [10].

The risk of injuries to healthcare workers is subjective to a range of factors, including the type of needle and other sharp objects used, alongside their safety systems, also depend on the number of patients and the precautions the healthcare workers observe while dealing with these patients [11]. Physicians, nurses, laboratory technicians, and those involved in medical waste management face a heightened risk of sharp object injuries in various stages of patient care, including screening, diagnosis, treatment, and monitoring. Additionally, the risk persists during the process of managing medical waste [12].

In Jordan, limited research has focused on sharp object injuries, with most data included from studies concentrating on practicing nurses or nursing students [13-18]. However, there is a dearth of studies examining sharp object injuries among healthcare workers other than nurses in Jordan, and none have delved into the practices and predisposing factors related to sharp object injuries among these HCWs [13,14]. Consequently, further research is necessary to comprehend the causes behind the high sharp object injury rate and the insufficient knowledge of safety practices and preventive guidelines.

Given the scarcity of similar studies in Jordan and the limited attention to HCWs' safety concerns in public hospitals, there is a pressing need for a comprehensive, informative, and precise research endeavor that can yield valuable insights and potential preventive strategies.

Therefore, this study aims to investigate the impact of sharp object injuries on our co-workers, underlying causes, reporting practices, potential consequences causes of needlestick injuries among hospital co-workers, and to highlight perspectives and preventive imperatives.

## Materials and methods

Ethics

Patient incidence reports from the preventive medicine database over 5 years (January 2018- January 2023) were analyzed. This study was registered by the institutional ethics committee, Royal Medical Services (IRB:11/2/2024), on the 12th of February 2024. As this study was a retrospective analysis, the requirement for patient consent was waived. All the participants’ data handling was accomplished according to the Declaration of Helsinki (2013) and the Health Insurance Portability and Accountability (HIPAA) Acts.

Participants

The comprehensive compilation of sharp injury reports involving Healthcare Workers is meticulously documented through the utilization of incident management protocols derived from the Department of Preventive Medicine at the Royal Medical Services. Throughout the designated study duration, our investigation delved into a retrospective analysis of incidents related to sharp object injuries, meticulously examining the reports submitted by healthcare workers. This examination encompassed a diverse spectrum of HCWs, spanning across various hospital departments, thereby guaranteeing a robust representation that reflects the multifaceted nature of healthcare practices. The inclusion of healthcare professionals from different specialties and roles enhances the depth and breadth of our study, providing valuable insights into the prevalence and patterns of sharp injuries in a comprehensive healthcare setting. The diverse range of participants ensures that our findings are applicable and informative across a wide array of healthcare disciplines, contributing to a more nuanced understanding of the challenges faced by healthcare workers in their daily tasks.

Study design

This retrospective institutional-based cross-sectional chart analysis was carried out by thoroughly examining every sharp object injuries report sheet. The data extraction process involved a comprehensive review of these reports, ensuring that every pertinent detail was accurately captured for subsequent in-depth analysis. Our study ambitiously spanned the entirety of reported cases that transpired between the years 2018 and 2023, allowing us to provide a comprehensive and contemporary overview of sharp object injuries within the institutional setting. The wealth of collected data encompassed a myriad of crucial information, including but not limited to age, gender, occupation, departmental affiliation, the precise location of injury, and the nature of the procedure or task being undertaken at the time of the incident. This multifaceted approach in data collection not only facilitates a nuanced exploration of the various factors associated with sharp injuries but also ensures a robust foundation for drawing comprehensive conclusions regarding the patterns, trends, and potential risk factors associated with these incidents in the institutional healthcare context.

Statistical analysis

Data was inputted into a Microsoft Excel spreadsheet, and SPSS Statistics Version 28.0 (IBM, Armonk, New York, USA) was utilized for analysis. Categorical data were represented through frequencies and percentages, while descriptive statistics like mean and standard deviation were employed for scale data. The Chi-square test evaluated associations within categorical data, and the independent t-test assessed mean variations in scale data between groups. Fisher exact tests were applied for preliminary investigations to understand how demographic variables affected participants’ perceptions of the healthcare system and their quality of life. Significance was determined at an Alpha level of 0.05.

## Results

A total of 146 self-reported hospital co-workers were included in the study, providing a comprehensive overview of the prevalence and characteristics of sharp object injuries. Within the final cohort, 52.73% of the participants were male (77/146), with an average age at diagnosis of 38.6±7.87 years (ranging from 20 to 52 years). Conversely, females comprised 47.27% of the cohort population (69/146) and had an average age at diagnosis of 34.73±6.73 years (ranging from 19 to 47 years). The age group 20-29 years was the most prominent age group, statistical analysis of age and gender data revealed significant differences as shown in Table 1.

**Table 1:** Comparison of sharp object injuries by location of injury to independent variables of healthcare workers in our center OR = operating room; Lab = laboratory; OP = outpatient; *= statistically significant.

Variable	n (%)						P value
	OR	Lab	Wards	OP clinic	House keeping	Other	
Age in years							
<20	0 (0)	0 (0)	0 (0)	0 (0)	0 (0)	1(100)	0.006*
20–29	5 (7.04)	11 (15.50)	27 (38.03)	13 (18.31)	9 (12.68)	6 (8.45)	
30-39	19 (33.33)	13 (22.81)	10 (17.54)	3 (5.26)	8 (14.04)	4 (7.02)	
40-49	3 (20)	2 (13.33)	3 (20)	4 (26.67)	2 (13.33)	1 (6.67)	
>50	1 (50)	0 (0)	0 (0)	0 (0)	1 (50)	0 (0)	
Gender							
Male	23 (29.87)	8 (10.38)	19 (24.67)	6 (7.79)	9 (11.68)	12 (15.58)	<0.001*
Female	3 (4.35)	16 (23.19)	26 (37.68)	4 (5.80)	19 (27.53)	1 (1.45)	
Job title							
Physician	20 (48.78)	5 (12.20)	10 (24.39)	6 (14.63)	0 (0)	0 (0)	<0.001*
Nurse	11 (28.94)	0 (0)	21 (55.26)	6 (15.79)	0 (0)	0 (0)	
Lab technician	0 (0)	22 (100)	0 (0)	0 (0)	0 (0)	0 (0)	
Housekeeping	5 (17.86)	7(25)	13 (46.43)	1(3.57)	2 (7.14)	0 (0)	
Paramedics	7 (58.33)	0 (0)	5 (41.67)	0 (0)	0 (0)	0 (0)	
Other	0 (0)	0 (0)	0 (0)	0 (0)	0(0)	5 (100)	
Task during which injury occurred							
Surgical intervention	18 (100)	0 (0)	0 (0)	0 (0)	0 (0)	0 (0)	<0.001*
Medical waste management	5 (15.63)	3 (9.38)	2 (6.25)	8 (25)	14 (43.75)	0 (0)	
Medication administration	4 (26.67)	0 (0)	8 (53.33)	3 (20)	0 (0)	0 (0)	
Histopathology preparation	0 (0)	7 (100)	0 (0)	0 (0)	0 (0)	0 (0)	
Needle recapping	19 (35.85)	4 (7.55)	21 (39.62)	6 (11.32)	0 (0)	3 (5.66)	
Unusual occurrence	1 (4.76)	0 (0)	4 (19.04)	7 (33.33)	1 (4.76)	8 (38.09)	
Site of injury							
Right hand	8 (5.48)	10 (6.85)	16 (10.96)	7 (4.79)	4 (2.74)	2 (1.37)	0.002*
Left hand	13 (8.90)	10 (6.85)	20 (13.70)	29 (19.86)	5 (3.42)	6 (4.11)	
Left leg	0 (0)	0 (0)	0 (0)	0 (0)	2 (1.37)	4 (2.74)	
Right leg	3 (2.05)	0 (0)	0 (0)	0 (0)	4(100)	3 (2.05)	

**Figure 1:**
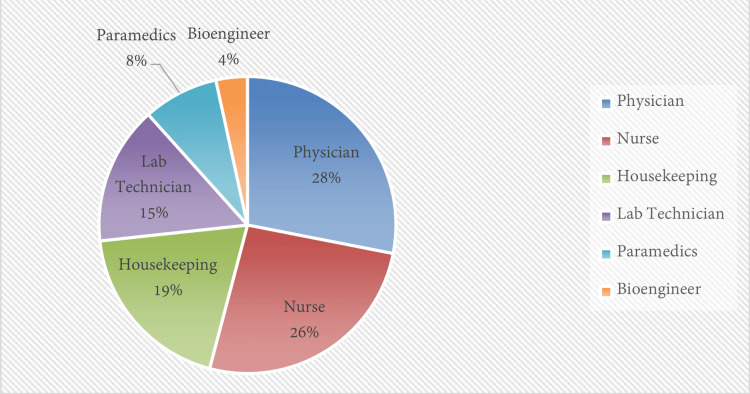
A chart showing the reported needlestick injuries in various occupations.

The overall prevalence of sharp object injuries was 11.83%, indicating that a sizable portion of HCWs is at risk of exposure to bloodborne pathogens due to sharp object injuries. Among the different professional categories, Physicians constituted the majority of sharp object injuries reported victims in 41 cases (28.08%), followed by nurses in 38 cases (26.02%), and housekeeping staff members in 28 cases (20.54%). In contrast, lab technicians reported 22 cases, constituting (19.18%), and paramedics staff reported sharp object injuries in 12 cases (8.22%) (Figure 1). Statistical analysis of the profession’s data revealed significant differences (P<0.001). 

**Figure 2:**
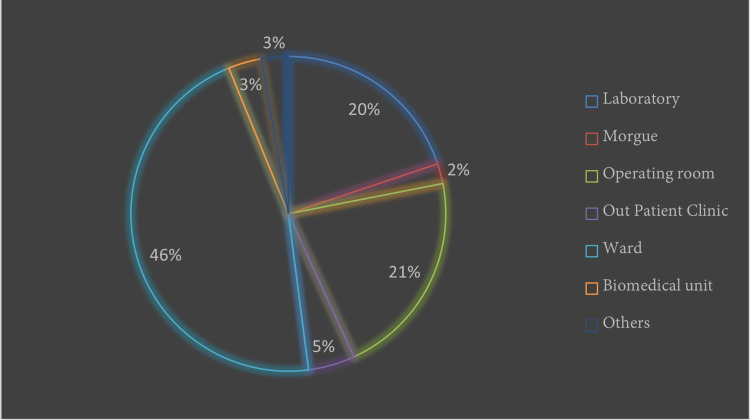
A chart showing the reported needlestick injuries in hospital sections.

This distribution reflects the varying levels of interaction with sharp medical instruments in distinct roles within the healthcare setting. Notably, sharp object injuries were most reported in wards, with 45.89% of all injuries occurring there, followed by operating room at 21.23%, while sharp object injuries in the laboratory registered 19.86% (Figure 2).

**Figure 3:**
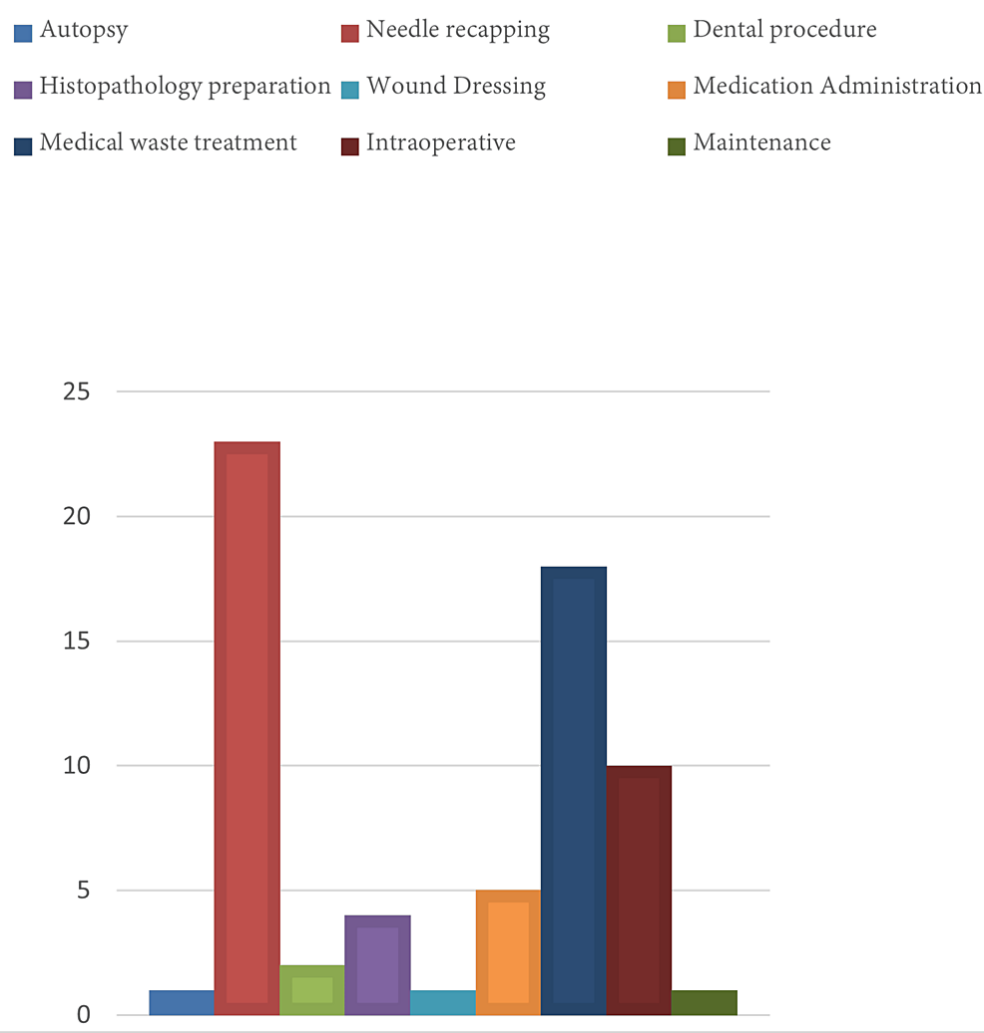
A chart showing the number of reported sharp object injuries in each procedure performed during which injury happened.

The leading procedures that caused sharp object injuries were identified as during needle recapping in 53 instances (36.30%), then followed by medical waste treatment in 32 cases (21.92%), while during intraoperative procedures, sharp object injuries were registered in 18 cases (12.33%), on the other hand during medication administration sharp object injuries reported in 15 cases (10.27%) (Figure 3).

The statistical analysis conducted on the data about the task or procedure during which sharp object injuries occurred, demonstrated a significant correlation (P<0.001)

Each cause contributes to a substantial proportion of injuries. All injured individuals reported the incident promptly. The left hand was the most affected body part, reported in 83 cases (56.84%). Analysis of body part injury data using statistical methods unveiled significant differences (P<0.002).

No seroconversions were documented within the reviewed cases during the study period.

## Discussion

Healthcare workers hold a vital role in delivering medical care, but they encounter various job-related dangers, notably needlestick and sharp injuries, which pose a significant risk. These incidents happen when healthcare workers accidentally puncture or cut themselves with contaminated medical sharp tools, especially needles, putting them in danger of bloodborne infections [19,20]. While precautions and safer needle designs have decreased the frequency of these injuries, they still occur, although far less frequently than before. Understanding of these injuries increased following the identification of the human immunodeficiency virus (HIV) in the early 1980s. However, present concerns after a sharp object injury incident primarily focus on hepatitis B or hepatitis C rather than HIV. Regulations have been implemented to aid healthcare facilities manage such incidents, outlining the right protocols for handling sharp object injuries and starting post-exposure HIV prevention measures. The Centers for Disease Control and Prevention (CDC) has established a framework to guide healthcare professionals in deciding when to initiate antiretroviral therapy [10,21]. Despite improvements in infection control practices, injuries from sharp objects continue to be a persistent threat, causing physical, mental, and financial hardships for affected individuals and healthcare institutions [22].

Most of the research findings highlighted a higher prevalence of sharp object injuries among women compared to men [13,14,23,24]. This trend could be attributed to the significantly larger representation of female healthcare workers in hospitals, resulting in greater responsibilities for female nurses than their male counterparts [25,26]. Additionally, studies suggest that women are more susceptible to stress than men, and job-related stress was found to increase the likelihood of sharp object injuries by 7.3 times among healthcare workers [24,2,27-29]. Recent research corroborates these findings, indicating that women are more inclined to report injuries, undergo tests, and seek post-traumatic care following sharp object injuries. This inclination might stem from a heightened sensitivity to pain or concerns about bloodborne infectious diseases post-injury [30]. Paradoxically, in our study, men predominated the population with slight deference, the ratio was 1.12:1.

The majority of earlier research indicates that nurses experienced the highest rates of sharp object injuries. The estimated percutaneous injury rate per one hundred thousand inpatient days for nurses is 2.43 annually, which is ten times higher than the reported rates of 0.23 for medical doctors and 0.20 for technologists [13-15,18,23]. However, our findings during the study period showed that physicians were the most affected group.

Low-work-experienced individuals tend to be the most affected registered group in most studies, which is attributed to lack of experience [23,31-35]. Conversely, some reports showed that experienced and highly educated HCWs are more likely to be affected [31]. Our study showed the age group 20-29 years was the most affected group, which might be credited to the tenure of training, education, and experience.

Our results highlight that sharp object injuries predominantly occurred within hospital wards, which is in line with previous results [13,34,36]. A reasonable explanation stems from the heightened activity in this hospital section, particularly the constant influx of patients entering and exiting the ward. Furthermore, the ward routinely accommodates a significant volume of procedures necessitating the use of needles and sharp instruments. These procedures predominantly fall within the purview of nursing responsibilities. This could elucidate the notable association between injuries occurring in the medical ward and the occupation, age group, and left-hand injuries, traits commonly observed among young nurses engaged in a dynamic hospital setting.

 Studies showed that a significant majority of sharp object injuries affected the left hand (56.84%), among other parts that might be injured. A noteworthy observation considering it is not the dominant hand for most individuals. These findings are in line with other studies [13,23,37,38]. 

Despite consistent educational efforts, many staff members persist in recapping needles, perpetuating this behavior as a primary cause of numerous sharp object injuries. Multiple studies have consistently identified recapping as the most prevalent cause of sharp object injuries [17, 39-41]. Our findings revealed that among all hospital procedures, needle recapping ranked as the most frequent procedure (36.30%), during which sharp object injuries occurred, followed by medical waste treatment.

The findings of this study illuminate the need for targeted interventions to mitigate the incidence of sharp object injuries and safety measures for hospital co-workers. The high frequency of sharp object injuries among nurses could be attributed to their direct patient care responsibilities, including administering injections, drawing blood, and managing intravenous lines. These tasks involve frequent interaction with sharp medical instruments and are performed under time constraints, increasing the risk of accidental injuries. Moreover, the concentration of sharp object injuries in specific departments suggests that the risk is not evenly distributed across the hospital, indicating a need for tailored prevention strategies in those areas. It is worth noting that despite the serious risks associated with sharp object injuries, a considerable portion of these injuries go unreported. The scanty reporting of sharp object injuries urges investigation into the contributing factors. Given that most occurrences are concentrated among younger individuals with minimal work experience, it emphasizes the need for targeted education and heightened awareness initiatives concerning safety matters within this demographic. Top of FormThis is concerning because unreported sharp object injuries can lead to missed opportunities for medical evaluation, timely intervention, and the initiation of post-exposure prophylaxis when necessary. To address this issue, hospital administrations must focus on fostering a culture of openness and non-punitive reporting, where HCWs are encouraged to promptly report any sharp object injuries, regardless of their perceived severity.

The observed variations of our study might stem from diverse factors such as differences in awareness levels, varying training opportunities, levels of exposure to sharp objects, as well as the availability and usage of protective devices. Recall bias and subtle methodological disparities among studies could also contribute to this variation. Prevalence rates can significantly differ across facilities due to varying standards, workload intensity, overcrowding issues, professional roles, skill levels, and the accessibility and utilization of resources. While lifetime prevalence might not offer a completely reliable estimate due to recall bias, we aimed to juxtapose our results with findings from other studies conducted elsewhere.

However, this study was subject to certain limitations. The data utilized relied on self-reporting, encompassing information solely from individuals acquainted with the implications of sharp object injuries on healthcare quality, potentially excluding those less informed about reporting sharp object injuries. Consequently, this dataset might not be fully representative of all sharp object injury incidents within the stipulated data collection period. Moreover, the study's scope was confined to a single healthcare institution in Jordan associated with the Royal Medical Services. Therefore, the findings necessitate validation through a broader dataset sourced from diverse public and private healthcare facilities across Jordan. Nevertheless, the study's outcomes contribute significantly to understanding the factors linked to sharp object injury occurrences, aiding the establishment of a national reporting system for sharp object injuries within Jordan's public healthcare sector. Importantly, such a system could serve as a model for implementation in other countries as well.

## Conclusions

Injuries caused by sharp objects persist as a significant danger for hospital employees, posing immediate harm and long-term health risks linked to bloodborne pathogens. This study emphasizes the crucial need for comprehensive preventive measures. Strategies must involve educating staff on safe practices, providing safety-engineered devices, and establishing efficient reporting systems.

The findings stress the continuous responsibility of healthcare institutions to prioritize staff safety by addressing the root causes of sharp object injuries and fostering reporting and prevention cultures. Through evidence-based interventions and a collective commitment to safety, hospital administrations can effectively decrease these injuries, safeguarding the health and livelihoods of their dedicated healthcare workforce. Underreporting reasons are diverse, encompassing factors like time constraints, fear of consequences, and the misconception of injury insignificance.
